# The diverse repertoire of ISG15: more intricate than initially thought

**DOI:** 10.1038/s12276-022-00872-3

**Published:** 2022-11-01

**Authors:** Ji An Kang, Yoon Jung Kim, Young Joo Jeon

**Affiliations:** 1 Department of Biochemistry, Chungnam National University College of Medicine, Daejeon, 35015 Korea; 2 Brain Korea 21 FOUR Project for Medical Science, Chungnam National University, Daejeon, 35015 Korea; 3 Department of Medical Science, Chungnam National University College of Medicine, Daejeon, 35015 Korea

**Keywords:** Ubiquitylation, Protein quality control

## Abstract

ISG15, the product of interferon (IFN)-stimulated gene 15, is the first identified ubiquitin-like protein (UBL), which plays multifaceted roles not only as a free intracellular or extracellular molecule but also as a post-translational modifier in the process of ISG15 conjugation (ISGylation). ISG15 has only been identified in vertebrates, indicating that the functions of ISG15 and its conjugation are restricted to higher eukaryotes and have evolved with IFN signaling. Despite the highlighted complexity of ISG15 and ISGylation, it has been suggested that ISG15 and ISGylation profoundly impact a variety of cellular processes, including protein translation, autophagy, exosome secretion, cytokine secretion, cytoskeleton dynamics, DNA damage response, telomere shortening, and immune modulation, which emphasizes the necessity of reassessing ISG15 and ISGylation. However, the underlying mechanisms and molecular consequences of ISG15 and ISGylation remain poorly defined, largely due to a lack of knowledge on the ISG15 target repertoire. In this review, we provide a comprehensive overview of the mechanistic understanding and molecular consequences of ISG15 and ISGylation. We also highlight new insights into the roles of ISG15 and ISGylation not only in physiology but also in the pathogenesis of various human diseases, especially in cancer, which could contribute to therapeutic intervention in human diseases.

## Protein function: Diverse roles of vertebrate protein

A protein called ISG15, found only in vertebrates, that can reversibly conjugate or bind to other proteins, tagging them for a wide array of activities, may be an attractive therapeutic target for various diseases. ISG15 is known to play roles in many processes, ranging from DNA repair to immune modulation, but its target range and molecular mechanisms remain poorly understood. Young Joo Jeon at Chungnam National University College of Medicine, Daejon, South Korea, and co-workers have reviewed current knowledge of ISG15, including its roles in disease. ISG15 activity is boosted in many cancers. They report that ISG15 can have different effects depending on which part of a protein it conjugates or binds to and its activation is likely to be tightly fine-tuned by many cues. Improving our understanding of its activities may help in many disease contexts.

## Introduction

Eukaryotic proteomes are tremendously sophisticated by protein processing and diversity of post-translational modifications (PTMs). Since the discovery of ubiquitin as a ubiquitous protein that is conjugated to other proteins in 1975^1^, more than a dozen human protein families referred to as ubiquitin-like proteins (UBLs) have been discovered that are structurally and evolutionarily related to ubiquitin, including interferon-stimulated gene 15 (ISG15), several paralogs of small ubiquitin-like modifier (SUMO), neural precursor cell expressed and developmentally downregulated 8 (NEDD8), human leukocyte antigen F locus (FAT10), ubiquitin-fold modifier 1 (UFM1), ubiquitin-related modifier 1 (URM1), autophagy-related protein 8 (ATG8), ATG12, Finkel-Biskis-Reilly murine sarcoma virus ubiquitously expressed (FUBI), and ubiquitin-like protein 5 (UBL5). UBLs commonly possess a β-grasp fold consisting of four- or five-stranded β-sheets, which partially wrap around a central helix^2^. The conjugation of these UBLs to target proteins or lipids is achieved through three sequential enzymatic reactions that are catalyzed by E1-activating enzymes, E2 conjugating enzymes, and E3 ligases. Additionally, the conjugation can be reversed by specific isopeptidases. Given that PTMs by UBLs play pivotal roles in the regulation of a large variety of cellular processes, including cell cycle control, DNA repair, intracellular trafficking, immune modulation, stress responses, and signal transduction, deregulation of UBL systems could be linked to a wide variety of human diseases, including cancers, neurodegenerative diseases, and immune diseases, which suggests that the components of UBL systems are attractive targets for the treatment of human diseases^3–6^.

ISG15 is the first UBL to be discovered^1,7^. Structurally similar to ubiquitin, ISG15 has two ubiquitin-like β-grasp domains separated by a short linker. Each domain is formed by four β-sheets and a single α-helix^8^. However, the primary sequences of these two ubiquitin-like β-grasp domains that correspond to the N- and C-terminal regions of ISG15 share only 29 and 31% identities with ubiquitin, respectively. The β-grasp fold of the C-terminal ubiquitin-like domain in ISG15 partially wraps around a short and flexible C-terminal tail terminating in diglycine residues through which ISG15 can be conjugated onto target proteins. Intriguingly, ISG15 is also present in an unconjugated intracellular or extracellular form. Extracellular unconjugated ISG15 plays a role as a cytokine to mediate interferon gamma (IFNγ) secretion^9–14^. Free intracellular ISG15 noncovalently associates with intracellular proteins and modulates their activities^15,16^.

In this review, we discuss recent advances in the mechanistic understanding and molecular consequence of ISG15 and its conjugation (ISGylation). We also highlight their physiological relevance and implications in human diseases.

## Characteristics of ISG15

### Properties of ISG15

Since ISG15 has the ability to cross-react with antibodies against ubiquitin, it was initially termed ubiquitin cross-reactive protein (UCRP)^17^. *Isg15* encodes an inactive precursor protein from which eight amino acids are cleaved off at the C-terminus and processed into its mature 17-kDa form to expose a carboxyl-terminal ‘Leu Arg Leu Arg Gly Gly’ (LRLRGG) motif that is required for its conjugation onto target proteins^18,19^.

**Fig. 1:**
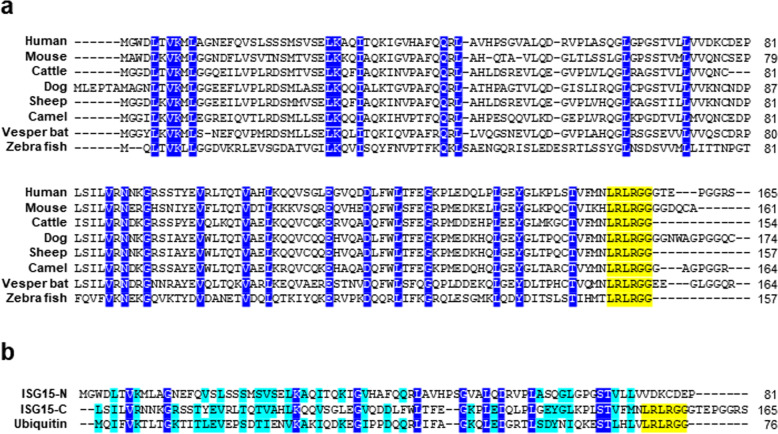
Amino acid sequence of ISG15. **a** The amino acid sequences of ISG15 from various species are aligned. **b** The amino acid sequences of ISG15 and ubiquitin are aligned. Similar amino acids are indicated by light blue, and identical amino acids are indicated by dark blue. The carboxyl-terminal LRLRGG motif is indicated in yellow.

ISG15 orthologs have been found only in vertebrates. Unlike the almost 100% cross-species conservation of ubiquitin, cross-species conservation of ISG15 is relatively low, hovering ~50% even among mammals. Chimpanzee, mouse, and opossum share 98, 63, and 42% conservation with humans, respectively (Fig. 1a)^20^. Moreover, the diversity of ISG15 amino acid composition among species has been suggested to influence the tertiary structure of ISG15 in different species^21–25^. ISG15 is composed of two tandem ubiquitin-like domains that have sequence homology with ubiquitin (Fig. 1b). The LRLRGG hexapeptide sequence is significantly conserved in the C-terminal domain of ISG15. While these two ubiquitin-like domains that correspond to the N- and C-terminal regions of ISG15 share only 29 and 31% sequence identities with ubiquitin, respectively, both of the N- and C-terminal domains show a striking similarity in their tertiary structures to ubiquitin and display comparable and distinct areas of electrostatic surface potentials with ubiquitin^8,26,27^. Interestingly, the solvent-exposed N-terminal domain promotes the transfer of ISG15 from the E2 conjugating enzyme to the target protein, and the C-terminal domain is critical for E1-activating enzyme-mediated activation of ISG15 and formation of thioester bond with ISG15.

### Expression of ISG15

ISG15 is robustly induced by IFNs, interleukin 1 beta (IL-1β), pathogenic infection, lipopolysaccharides (LPS), retinoic acid (RA), hypoxia, experimental stroke traumatic brain injury, or DNA-damaging stresses^4,5,18,20,28–40^. ISG15 is primarily induced by Type I IFNs. *Isg15* has two interferon-stimulated response elements (ISREs) in its promoter region^41^. A number of IFN regulatory factors (IRFs), including IRF3 and IRF9, bind to the ISRE^41,42^. IRF9 interacts with the signal transducer and activator of transcription 1 (STAT1) and STAT2 and forms the IFN-stimulated gene factor 3 (ISGF3) complex, resulting in ISG15 induction. IRF3 forms a complex with CREB-binding protein (CBP)/p300 coactivators for the induction of ISG15^43,44^. ISG15 expression is induced by PU.1, a member of the Ets family of transcription factors. *Isg15* contains a PU.1 binding site in its promoter region, which overlaps with the ISRE sequence. PU.1 with IRF4 or IRF9 synergistically induces ISG15^45^. ISG15 is also induced by Type II and III IFNs^46,47^.

ISG15 is induced by bacterial and viral infections. Bacterial and viral infections activate IRF3 and ISGF3, which are involved in IFN signaling^48^, resulting in ISG15 induction. ISG15 is also induced by LPS. When macrophages are stimulated by LPS, ISG15 can be detected as early as 1 h, and its level becomes maximal at ~4 h^49^. In type I IFN receptor R1 knockout mice and cells, ISG15 expression is attenuated upon treatment with LPS or viral infections^50,51^, suggesting that activation of type I IFN signaling by bacterial and viral infections induces ISG15 expression.

RA induces ISG15 in acute promyelocytic cells^40,52^. The RA-mediated accumulation of ISG15 occurs in RA-sensitive leukemic cells but not in RA-resistant cells, and the pattern of accumulated ISG15 conjugates is similar to that observed by type I IFN treatment. Interestingly, IRF1 and (2′–5′) oligoadenylate synthetase are induced by RA^53,54^. RA treatment also leads to an increase in type I IFN secretion, and blockade of the type I IFN receptor with a neutralizing antibody inhibits ISG15 induction by RA, suggesting that RA elevates the level of ISG15 by stimulating cells to secrete IFNs.

Integrin adhesion-induced myocardin-related transcription factor-A (MRTF-A)-serum response factor (SRF) induces ISG15^55^, while kruppel-like factor 9 (KLF9)^56^ or cytochrome P450 1B1 (CYP1B1)^57^ inhibits ISG15 expression.

Noncoding RNAs have been demonstrated to regulate ISG15 expression. microRNA-138 (miR-138) decreases the mRNA level of *Isg15* in oral squamous carcinoma cells^58^. miR-370 associates with the 3’UTR in *Isg15* mRNA, downregulating ISG15 expression in hepatocellular carcinoma cells^59^. Inhibition of suppressor of cytokine signaling 3 (SOCS3) expression by miR-2909 upregulates *Stat1* and its downstream target *Isg15* in prostate cancer cells^60^. However, the mechanisms by which the expression of miRNAs is regulated are not fully defined.

Depletion of Bcl-2 associated athanogene 3 (BAG3) impairs ISG15 translation in pancreatic ductal adenocarcinoma (PDAC) cells, suggesting the role of BAG3 in the control of ISG15 expression^61^. Taken together, ISG15 expression can be tightly fine-tuned by intracellular and extracellular perturbations.

### ISG15 as a post-translational modifier

#### ISG15 conjugation machinery

**Fig. 2:**
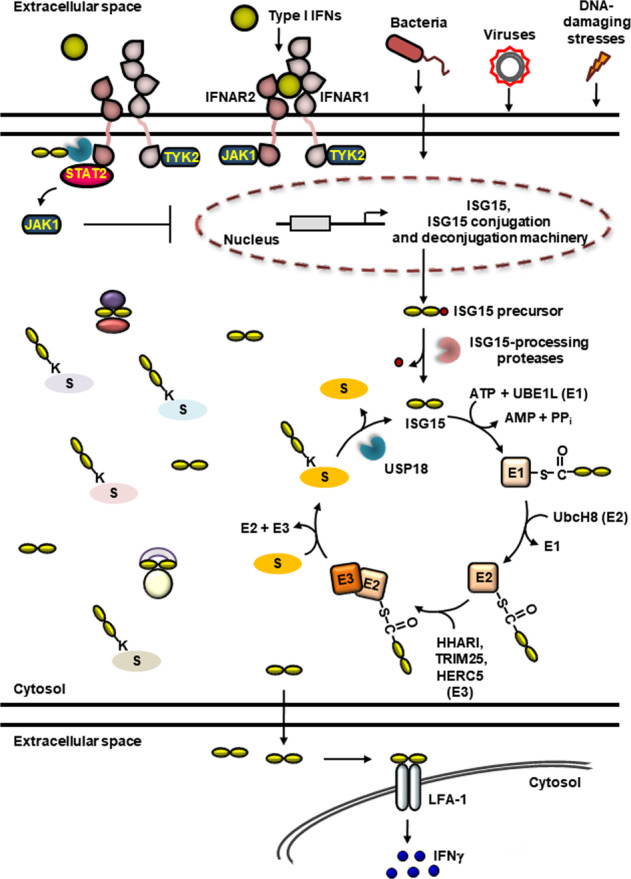
Multifaceted roles of ISG15 and its conjugation. The expression of ISG15 and enzymes involved in ISGylation and deISGylation is strongly induced by IFNs, viral and bacterial infection, and DNA-damaging stresses. ISG15 exists as an immature precursor and is proteolytically processed into its mature form, which leads to the exposure of a carboxyl-terminal LRLRGG motif that is essential for its conjugation to target proteins. Similar manner to ubiquitination, ISGylation utilizes a three-step enzymatic reaction. ISG15 is activated by the E1-activating enzyme UBE1L at the expense of ATP and subsequently bound to UBE1L via thioester bond. Following activation, ISG15 is transferred to the active-site cysteine of the E2 conjugating enzyme UbcH8 and then to a target protein with the aid of an E3 ligase, such as HHARI, TRIM25, or HERC5. USP18 reverses ISGylation by cleaving off ISG15 that is conjugated to target protein via isopeptide bond. Apart from a post-translational modifier, free intracellular ISG15 modulates not only the activity of proteins but also protein‒protein interactions, thereby resulting in the regulation of a large variety of biological processes. Type I IFN-induced USP18 acts as a negative feedback regulator of type I IFN signaling. USP18 decreases the cell surface-binding affinity of type I IFNs. Further, STAT2-mediated recruitment of USP18 to IFNAR2 competes with and displaces JAK1 from IFNAR2, which attenuates type I IFN signaling and suppresses downstream expression of ISGs. In humans, this process is dependent on the direct association of ISG15 with USP18. Free extracellular ISG15 functions as a cytokine for NK and T cells, where it binds to LFA-1 and modulates innate and adaptive immune responses.

Similar manner to ubiquitination, ISGylation of target proteins involves a three-step cascade of enzymes (Fig. 2). ISG15 coordinates with only five of the over 600 identified E1-activating-E2 conjugating-E3 ligase enzymatic members^62^. The first step of ISGylation is the activation of ISG15 through an ATP-dependent mechanism to form a thioester bond between the catalytic cysteine of the E1-activating enzyme UBE1L (UBA7) and the C-terminal glycine residue of ISG15^8,18^. Human UBE1L is a 112 kDa protein that possesses 45% amino acid sequence identity to the human ubiquitin-activating E1 enzyme UBE1. UBE1L expressed in baculovirus forms a thioester bond with ISG15 but not with ubiquitin, suggesting that UBE1L is an ISG15-specific E1 enzyme. UBE1L has a C-terminal ubiquitin-fold domain that is required not only for the transfer of ISG15 from UBE1L to the E2 conjugating enzyme UbcH8 (UBE2L6) or its murine counterpart UbcM8 but also for the binding of UBE1L to UbcH8. Following activation, ISG15 is transferred from UBE1L to an active-site cysteine residue on UbcH8 via transthiolation^63,64^. While UbcH8 is able to participate in ubiquitination as an E2 conjugating enzyme in vitro, UbcH8 has a significantly higher affinity for UBE1L over UBE1, suggesting that UbcH8 is an ISG15-specific E2 enzyme in vivo^65^. Finally, E3 ligases, involving a really interesting new gene (RING) E3 ligase tripartite motif-containing protein 25 (TRIM25; Efp)^66,67^, RING-between-RING (RBR) E3 ligase human homolog of Ariadne (HHARI)^68^, and human homologous to E6AP C-terminus (HECT) and RLD domain containing E3 ligase 5 (HERC5) or its murine counterpart HERC6^69,70^, facilitate the conjugation of ISG15 to target proteins. Of note, whereas HHARI and TRIM25 exhibit some substrate specificity, HERC5 shows broadness and promiscuousness in substrate specificity.

#### ISG15 deconjugation machinery

*Ubiquitin specific peptidase 18* (*Usp18*) was originally referred to as *ubiquitin processing protease 43* (*Ubp43*) since it encodes UBP with a calculated molecular weight of 43 kDa. *Usp18* is remarkably induced by type I and III IFNs^71–74^, polyinosinic:polycytidylic acid (poly I:C)^50^, tumor necrosis factor alpha (TNFα)^75^, LPS^50,75^, or genotoxic stresses^71,76^. *Usp18* is also upregulated upon pathogenic infection^74,77–79^. USP18 possesses cysteine and histidine boxes, which are significantly conserved in ubiquitin-specific proteases (USPs) and essential for enzymatic activity^80–82^.

USP18 is the major deubiquitinating enzyme (DUB) that reverses ISGylation^21,71,81,83,84^ (Fig. 2). Analysis of the specificity of USP18 using ^125^I-labeled ubiquitin and UBLs has demonstrated that USP18 dominantly cleaves off ISG15 from ISGylated proteins^71^. In contrast to the original findings, it has been suggested that USP18 can also recognize ubiquitin and remove ubiquitin from its conjugated proteins in a specific context^85,86^.

### ISG15 as a free intracellular or extracellular protein

#### Free intracellular ISG15

Free intracellular ISG15 can associate with intracellular proteins in a noncovalent manner, resulting in the modulation of protein‒protein interactions and functions of its interacting partners (Fig. 2). ISG15 binds to NEDD4 and impairs its activity, which inhibits VP40 ubiquitination, suggesting the antiviral activity of free intracellular ISG15 through blockage of the E3 ligase activity of host NEDD4^16^. The association of ISG15 with USP18 interrupts the interaction of USP18 with S-phase kinase-associated protein 2 (SKP2), inhibiting the proteasomal degradation of USP18, which is essential for negative feedback regulation of IFN signaling and prevention of autoinflammation^15,87^.

#### Free extracellular ISG15

Free extracellular ISG15 has been suggested to have immunomodulatory functions, although the mechanistic understanding and biological functions of free extracellular ISG15 still remain to be explored (Fig. 2). ISG15 may not be secreted via the classical protein transport pathway since ISG15 is deficient in signal peptide for secretion^10,88^. Instead, ISG15 has been suggested to be localized in neutrophil granules and microvesicles for its secretion^89^. ISG15 can be secreted through exosomes originating from toll-like receptor 3 (TLR3)-activated human brain microvascular endothelial cells^90^ or apoptosis. Recently, the initial steps of extracellular ISG15 signaling have been demonstrated. Free extracellular ISG15 directly binds to cell surface receptor lymphocyte function-associated antigen 1 (LFA-1), which facilitates the activation of SRC family kinases (SFKs) and results in the release of IFNγ and IL-10 in natural killer (NK) cells and T lymphocytes^91^. Dimeric and multimeric forms of extracellular ISG15 have been suggested to be important for its cytokine activity and IL-1β production upon parasite infection^92^.

Free extracellular ISG15 has been reported to be secreted in several different cell types, including human primary monocytes, neutrophils, fibroblasts, and plasmablasts, in a type I IFN-dependent or IFN-independent manner^9,10,89,93^. ISG15 is detected in the serum of patients treated with IFNβ and of hepatitis B virus (HBV)-infected patients^10^. As a cytokine, ISG15 increases the cytotoxicity of LPS-stimulated primary monocytes, stimulates IFNγ production, induces NK cell proliferation, and promotes dendritic cell maturation^12–14,89,94^. In human monocytes, ISG15 promotes IL-10 production, which might be a useful biomarker for the determination of the severity of active tuberculosis^95^. ISG15 secretion in plasmablasts derived from patients with lupus erythematosus has been demonstrated, although it remains elusive whether free extracellular ISG15 has a protective or detrimental role in the pathogenesis of lupus erythematosus^93^. Interestingly, deficiency in extracellular ISG15 but not in intracellular ISG15 and its conjugation is linked to a decrease in IFNγ production by lymphocytes and aggravated susceptibility to mycobacterial disease in humans, suggesting the pivotal role of extracellular ISG15 in optimal antimycobacterial immunity^89^.

## Biological implications of ISG15 and its conjugation

ISG15 and its conjugation are implicated in a large variety of biological processes in a cell- and tissue-type-dependent manner. Recently, noncovalent interactome of ISG15 in human cells has been identified^96^, expanding the repertoire of ISG15. Moreover, several quantitative proteomics analyses, including enrichment and affinity purification, labeled ISG15 proteomics, and subtraction-based proteomics, have been performed to identify targets for ISGylation, an “ISGylome”, and to comprehensively demonstrate the role of ISGylation^97–101^. However, only a few of these targets have been validated, and even fewer have been functionally characterized.

### ISG15 and its conjugation in the DNA damage response

Genome integrity is continuously challenged by extrinsic and intrinsic perturbations. These DNA-damaging stresses cause DNA lesions that, if not repaired accurately, are capable of disturbing critical cellular processes. Inaccurate repair of DNA lesions can give rise to mutations and chromosomal abnormalities, which could lead to tumorigenesis, immunodeficiency, neurodegeneration, infertility, and premature aging, highlighting the importance of genome integrity for human health. To deal with DNA lesions, cells have evolved sophisticated and coordinated pathways, referred to as the DNA damage response (DDR). Notably, proteomic studies have revealed ISG15 as a pivotal interactor of a considerable number of potential targets involved in DDR and maintenance of genome integrity^4,28,32,70,97,98,102^.

Telomeres function as protective chromosome ends to ensure genome stability. *Isg15* is located at 1p36.33, the subtelomeric end of chromosome 1p, whose expression is regulated by telomere length in human cells^103^. Furthermore, telomere shortening modulates the expression of specific genes through the telomere position effect over long distances (TPE-OLD)^104^. *Isg15* has emerged as a gene modulated through TPE-OLD, in which ISG15 expression is inversely correlated with telomere length, suggesting that ISG15 monitors telomere length and transduces signals for initiation of DDR, contributing to genome stability^103–105^.

Translesion DNA synthesis (TLS) is a DNA damage tolerance process that allows cells to bypass DNA lesions while tolerating the repair of DNA lesions at a later stage, thereby forestalling the collapse of replication forks. Ultraviolet-induced proliferating cell nuclear antigen (PCNA) ISGylation plays a pivotal role in TLS termination, thereby preventing excessive mutations^28^. An increase in replication fork speed above a threshold results in DNA damage and genomic instability, whereby faster-replicating forks have insufficient time to recognize and repair damaged DNA. Recently, it has been proposed that ISG15 upregulation increases replication fork speed and leads to DNA damage and genome instability, which modulates cellular sensitivity to DNA damage-inducing agents^106^. Functional interaction of ISG15 with RECQ1 independent of ISGylation regulates RECQ1 by unleashing its reversed fork restart activity. Further, recent studies have demonstrated that defects in replication fork processing lead to the accumulation of cytosolic DNA and transactivate innate immune response genes^107^. DNA damage triggers innate immune responses through the accumulation of cytoplasmic ssDNA or dsDNA, which activates cyclic guanosine monophosphate (GMP)-adenosine monophosphate (AMP) synthase (cGAS)-stimulator of interferon genes (STING), a major sensor for cytosolic ssDNA or dsDNA^108^. cGAS senses cytosolic DNA and synthesizes secondary messenger 2’,3’-cyclic GMP-AMP. Synthesized 2’,3’-cyclic GMP-AMP is detected by STING, which promotes IRF3 activation for type I IFN production and IFN-related DNA damage resistance signature (IRDS) gene expression. The majority of IRDS genes are a subgroup of ISGs. Interestingly, *Isg15* and *Usp18* belong to IRDS genes. Intriguingly, IRDS genes are upregulated in diverse cancer types and associated with DNA-damaging chemo- and radiotherapy^109^. Signatures of IRDS genes are found in cancer patients showing resistance to DNA damage-inducing therapies^110–112^. In breast cancer, IRDS genes, including *Isg15, Stat1, Mx1, Oas1, Ifit1, Ifit3*, and *Ifi44*, are associated with resistance to therapies, and their downregulation resensitizes triple-negative breast cancer (TNBC) cells to chemo- and radiotherapy^113^, suggesting that targeting IRDS genes in cancer could be a promising approach to increase therapeutic efficacy.

### ISG15 and its conjugation in protein translation

**Fig. 3:**
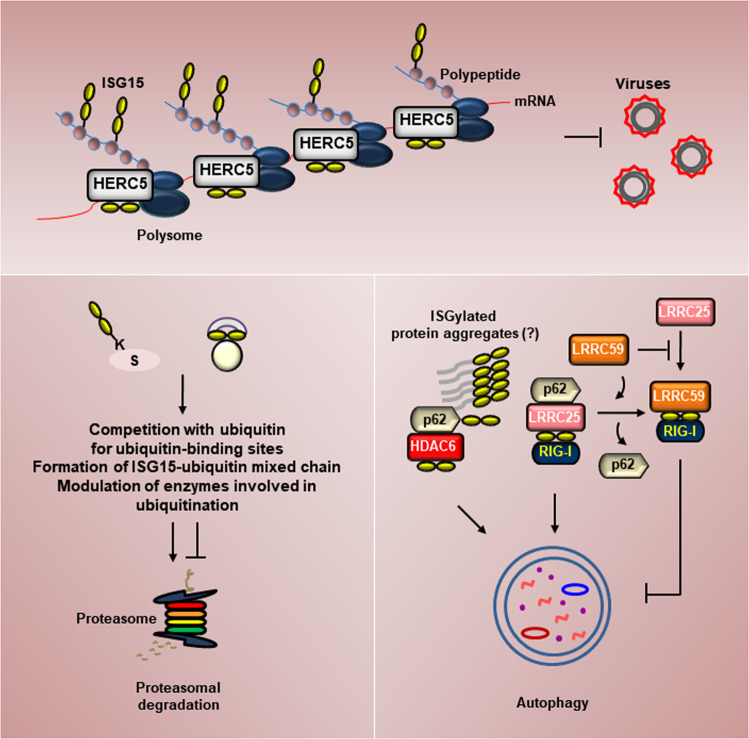
ISG15 and its conjugation in proteostasis. The E3 ligase HERC5 associates with polysomes and facilitates ISGylation of newly synthesized proteins in a cotranslational manner, which restricts newly synthesized nascent pools of proteins, including viral proteins. ISG15 and its conjugation positively or negatively regulate UPS by competing with ubiquitin for ubiquitin-binding sites on a target protein, forming ISG15-ubiquitin mixed chain, or modulating the activity of enzymes involved in ubiquitination. Free ISG15 associates with p62 and HDAC6, which augments p62-mediated aggresome formation and autophagic degradation under conditions of cellular stress. ISG15 also binds to LRRC25, p62, and RIG-I and subsequently mediates autophagy-mediated RIG-I degradation. LRRC59 binds to RIG-I associated with ISG15 and prevents its association with LRRC25, thereby leading to the inhibition of autophagy.

ISG15 can modulate protein synthesis not only by inhibiting global or mRNA-specific translation^68,114,115^ but also by suppressing limited protein translation^116^, which is largely associated with antiviral responses whereby translation of newly synthesized viral proteins is restricted. ISG15 acts as a cotranslational modulator by mediating the degradation of nascent viral or misfolded proteins. Polyribosome-associated HERC5 catalyzes broad ISGylation of newly synthesized proteins in a cotranslational manner, which limits newly synthesized nascent pools of proteins and facilitates antigen presentation on MHC class 1 molecules (Fig. 3)^117,118^. ISGylation of human papillomavirus (HPV) L1 capsid protein inhibits HPV16 infection, providing a basis for understanding that ISGylation restricts the translation of viral proteins and is implicated in the antiviral response. ISGylation of viral nucleoprotein (NP) suppresses viral RNA and protein synthesis, leading to a decrease in virus replication^119^. Coxsackievirus B3 (CVB3) 2 A protease ISGylation inhibits cleavage of host cell eukaryotic initiation factor eIF4G during CVB3 infection, which attenuates translational shut-off of host cells while suppressing internal ribosome entry site (IRES)-driven translation of the viral genome^120^, indicating the role of ISGylation in the subversion of virus-induced translational shut-off.

4EHP is ISGylated^68^, and ISGylated 4EHP possesses a higher affinity for m^7^GTP than its unmodified form and suppresses specific mRNAs translation. Protein kinase R (PKR) ISGylation activates PKR, resulting in eIF2α phosphorylation and downregulation of global protein translation^114^. However, further investigation is required to understand the implication of ISG15 in the control of translation beyond the antiviral context.

### ISG15 and its conjugation in the ubiquitin‒proteasome system (UPS)

Many efforts have been made to define a link between ISGylation and ubiquitination (Fig. 3). It has been reported that proteasomal inhibition results in a marked increase in ISG15 conjugates, which is the first link demonstrating the relationship of ISGylation with proteasomal degradation^121^.

Upregulation of ISGylation inhibits proteasomal degradation and contributes to progressive neurodegeneration in ataxia telangiectasia (A-T) patients, suggesting that ISGylation could affect the turnover of ubiquitinated proteins^122^. IRF3 ISGylation attenuates ubiquitination and proteasomal degradation of IRF3^123^. Mechanistically, upon viral infection, IRF3 ISGylation impairs the interaction of IRF3 with the peptidyl-prolyl *cis-trans* isomerase NIMA-interacting 1 (PIN1) and antagonizes the ubiquitination and degradation of IRF3, which results in persistent IRF3 activation and antiviral responses. STAT1 ISGylation inhibits the ubiquitination and degradation of STAT1, maintaining its function^124^. ISG15 can inhibit proteasomal degradation via competition with ubiquitin for conjugation sites on target proteins^125^. ISGylation of ubiquitin at lysine 29 results in the formation of ISG15-ubiquitin mixed chains^126^, which dampens cellular turnover of ubiquitinated proteins, suggesting an unanticipated interplay between ISGylation and ubiquitination for maintaining proteostasis. However, the field of ISG15-ubiquitin hybrid chains is less explored, and hybrid chain recognition domains still need to be identified. Several studies have reported that ISG15 impairs the activity of enzymes involved in ubiquitination. ISG15 specifically binds to NEDD4 and impairs the interaction of NEDD4 with ubiquitin-E2 molecules, thus preventing the further transfer of ubiquitin from E2 to NEDD4^16,127^. Ubc13 ISGylation suppresses the catalytic activity of Ubc13 as a ubiquitin-E2 conjugating enzyme^97^.

On the contrary, ISG15 has been demonstrated to facilitate proteasomal degradation. Forkhead box O3 (FOXO3a) ISGylation leads to its degradation^128^. Under hypoxia, hypoxia-inducible factor 1 alpha (HIF1α) is ISGylated, which facilitates its ubiquitination and degradation^33^. β-catenin ISGylation leads to its ubiquitination and proteasomal degradation^129^. ISGylation has been demonstrated in the regulation of RA-inducible gene I (RIG-I) and melanoma differentiation-associated protein 5 (MDA5)-mediated innate immunity. ISGylation of RIG-I and MDA5 promotes lysine 48-linked ubiquitination by ring finger protein 125 (RNF125) and proteasomal degradation of RIG-I and MDA5^130^. Interestingly, MDA5 ISGylation upon viral infection plays an analogous role to lysine 63-linked ubiquitination, leading to MDA5 oligomerization and antiviral immunity^131^. ISGylation of the carboxyl terminus of hsp70-interacting protein (CHIP) facilitates its enzymatic activity, which leads to a decrease in c-Myc and attenuates tumor growth^132^. Similarly, Parkin ISGylation facilitates its enzymatic activity^133^, indicating the close relationship between ISGylation and ubiquitination.

In healthy cells, misfolded or dominant-negative p53 is preferentially ISGylated by HERC5, which results in proteasomal degradation of misfolded p53, facilitating wild-type p53 activity^134^. ISG15 depletion results in the accumulation of misfolded or dominant-negative p53, which inhibits p53 and leads to a decrease in DNA damage-induced senescence and an increase in cell proliferation. In transformed cells, native as well as misfolded p53 might be ISGylated, which leads to proteasomal degradation of p53 and an overall decrease in p53 activity, thereby promoting tumor progression^135^. Collectively, these findings indicate the distinct roles of ISGylation in the control of p53 in a context-dependent manner.

To summarize, the relationship between ISGylation and ubiquitination is somewhat controversial. Proteasome inhibitors, such as MG132 and lactacystin, have been reported not to affect ISGylation in the absence or presence of IFNs^99^. In contrast, proteasomal inhibition has been demonstrated to result in an increase in ISGylation^121^. Furthermore, general ubiquitination patterns between *Ube1L*^+/+^ and *Ube1L*^−/−^ cells are similar even after IFN treatment^136^. These controversial results suggest differential cell type- or context-dependent responses in the ISGylation-ubiquitination regulatory circuit. Therefore, it might be important to investigate conjugation preference, scope, and biological outcomes in vitro and in vivo models to decipher the ISGylation-ubiquitination regulatory circuit.

### ISG15 and its conjugation in autophagy

ISG15 has begun to emerge as an essential regulator in autophagy (Fig. 3). Autophagy is a process by which cytoplasmic constituents, including organelles, aggregates, and proteins, are degraded by lysosomes. Differing from initial speculation that autophagy is a nonselective catabolic system, it has been demonstrated that chaperones and other cargo-recognition molecules, including sugar- or lipid-based signals, ubiquitin, and UBLs, confer a selective nature on this catabolic process^137^. Persistent upregulation of ISGylation induces aberrant autophagy upon genotoxic stress in certain pathological circumstances^138^. ISG15 facilitates p62-mediated aggresome formation and aggresome degradation via aggrephagy, a selective autophagy-clearing protein aggregates^139^. ISG15 colocalizes with p62 and histone deacetylase 6 (HDAC6) in cytosolic inclusion bodies, which leads to the recruitment of misfolded proteins to dynein motors for their transport to aggresome and autophagosome-lysosome fusion^140,141^. Recently, it has been reported that NEMO ISGylation is essential for the recruitment of autophagy machinery to attenuate RANKL signaling^142^. TSG101 ISGylation promotes degradation of TSG101 via autophagy and leads to impairment of exosome secretion, suggesting that ISG15 is a novel UBL in the control of exosome production^143^. In response to various stimuli, particular proteins and nucleic acids, in addition to cytosolic contents, are selectively sorted into exosomes^144,145^. The mechanisms by which exosome composition and content are fine-tuned remain elusive. Given that exosomes are key mediators of cell-to-cell communications in a variety of processes, including immune responses^146,147^, tumorigenesis^148,149^, and neuron survival, it might be important to decipher the role of ISG15 in the modulation of cell-to-cell communications mediated by exosomes.

The role of ISG15 in immune responses is associated with autophagy. ISG15 is involved in the recruitment of p62, NDP52, and LC3 to parasitophorous vacuoles (PVs), contributing to IFNγ-restricted parasite growth^150^. Upon RNA virus infection, leucine-rich repeat-containing protein 25 (LRRC25) interacts with ISG15-associated RIG-I, which triggers the association of RIG-I with p62, resulting in autophagic degradation of RIG-I and termination of the antiviral response, indicating that the ISG15-RIG-I-LRRC25 axis forms a negative feedback loop to maintain the balance of type I IFN signaling^151^. LRRC59 binds to RIG-I associated with ISG15 and prevents its association with LRRC25, resulting in stronger antiviral responses^152^. Herpes simplex virus (HSV) infection of trigeminal ganglia (TG) neurons facilitates the formation of autophagosomes decorated with ISG15 and p62, which is linked to antiviral signaling, representing a neuronal response to HSV infection. Autophagy is impaired in ISG15-deficient macrophages, leading to disruption of the removal of damaged and dysfunctional mitochondria^153^. Mitochondrial oxidative phosphorylation and the production of reactive oxygen species (ROS) are lower in *Isg15*^*−/−*^ bone marrow-derived macrophages (BMDMs) following IFN treatment than in *Isg15*^*+/+*^ BMDMs, indicating the role of ISG15 in the control of mitochondrial dynamics.

On the contrary, Beclin 1 ISGylation inhibits autophagy^154^. Beclin 1 ubiquitination disrupts the interaction of Beclin 1 with its negative regulator BCL-2 and promotes autophagy^155^. Beclin 1 ISGylation blocks its ubiquitination in the late period of IFN treatment and attenuates Beclin 1-promoted autophagy^154^, suggesting that long-term treatment with IFN inhibits autophagy through Beclin 1 ISGylation, while a transient response to IFN facilitates autophagy. TRIM21 ISGylation enhances its enzymatic activity and facilitates lysine 63-linked ubiquitination of TRIM21 and p62, preventing p62 oligomerization and subsequent localization to the autophagosome^156^.

Increasing evidence has suggested that mitophagy is defective in neurons of patients with various neurodegenerative diseases, including Alzheimer’s disease, Parkinson’s disease, A-T, and amyotrophic lateral sclerosis (ALS). Importantly, the level of ISG15 is constitutively upregulated in mitophagy-defective A-T and ALS, suggesting ISG15 as a biomarker for defects in mitophagy and neuronal injury^122^. Suppression of ISG15 and ISGylation leads to restoration of mitochondrial dynamics and reduction in oxidative stress, resulting in re-establishment of mitophagy in A-T cells^157^. On the contrary, ISG15 inhibits ubiquitination and proteasomal degradation of proteins in A-T cells, which facilitates basal autophagy as a compensatory mechanism for protein turnover in A-T cells and results in neuroprotection^122,138^.

Although ISG15 and its conjugation have emerged as important players in selective autophagy, the exact mechanisms remain to be explored. Further functional analyses are critical for the determination of ISG15 and ISGylation in autophagy, involving the identification of targets for ISGylation, determination of autophagy receptors that link ISG15-decorated cargos and autophagy, dissection of mechanistic roles of ISGylation during autophagy and involvement of ISGylation in multiple layers of regulatory mechanisms that control dynamic autophagy networks.

## Physiological and pathophysiological implications of ISG15 and its conjugation

### ISG15 and its conjugation in tissue differentiation

ISG15 and ISGylation have been demonstrated to function in normal tissue differentiation, especially in placental and fetal development^158–163^. Murine endometrial proteins are ISGylated during early pregnancy. ISG15 and its conjugates are present in implantation sites during mid- to late gestation^163^. USP18 deletion leads to an increase in ISG15 and its conjugates at the feto-maternal interface and results in fetal death in a mixed genetic background^163^, suggesting the requirement of USP18 for normal ISG15 expression and fetal development. ISG15 abundance in the human placenta and the maximal expression of ISG15 in the first and second trimesters of pregnancy reveal its implications for placental and embryo development, fetal growth, and potential defense mechanisms against infections^162^. A study of reproductive phenotype using *Isg15*^*−/−*^ mice indicated that 50% of fetuses died between 7.5 and 12.5 d postcoitum (dpc) in *Isg15*^*−/−*^ female mice when mated with *Isg15*^*−/−*^ male mice^35^. Embryo mortality occurs in pregnant *Isg15*^−/−^ female mice and is exacerbated by environmental insults such as maternal hypoxia that may not be counteracted in pregnant *Isg15*^−/−^ mice, which might result from impaired early decidualization, vascular development, and formation of the labyrinth^164^.

ISGylation might play an important role in the differentiation of monocytes, erythroid elements, and dendritic cells (DCs)^80,165,166^. Mutations in *Isg15*, *Ube1L*, and *Usp18* could lead to abnormal phenotypes associated with immunity and hematopoiesis^15,89,165–167^. It has been reported that RIG-I binds to *Trim25* mRNA in acute promyelocytic leukemia (APL) cells following all-*trans* RA (ATRA) treatment, which upregulates TRIM25 expression and induces ISGylation, contributing to myeloid differentiation and maturation^168^.

### ISG15 and its conjugation in metabolic reprogramming

ISG15 and ISGylation have recently been linked to metabolism. Systemic identification of endogenous ISG15 substrates in the liver following infection with *Listeria monocytogenes* revealed that targets for ISGylation are enriched in proteins that are involved in cellular metabolic processes^101^. Enhanced ISGylation promotes basal and infection-induced autophagy via mammalian target of rapamycin (mTOR), WD repeat domain, phosphoinositide interacting 2 (WIPI2), activating molecule in Beclin-1-regulated autophagy (AMBRA1), and Ras-related protein (RAB7) modifications, suggesting that ISGylation of metabolic enzymes temporally reprograms organismal metabolism following infection in the liver. ISG15 enhances oxidative capacity and gluconeogenesis during CVB3 infection^169^. Increased expression of ISG15 and its conjugation in pancreatic cancer stem cells (PaCSCs) is essential for maintaining the metabolic plasticity of PaCSCs^170^. ISG15 depletion leads to decreased ISGylation in mitochondria accompanied by increased accumulation of dysfunctional mitochondria, reduced oxidative phosphorylation, and impaired mitophagy, disrupting mitochondrial metabolism and downregulating PaCSC stemness. ISG15, as a downstream target of IRF3, is conjugated to glycolytic enzymes, which decreases lactate production and reprograms adipocyte metabolism, thereby mediating the effect of IRF3 on thermogenesis^100^. ISG15 depletion in vivo promotes adipose thermogenesis and protects mice from high-fat diet-induced obesity and glucose intolerance, suggesting the role of ISG15 in the modulation of glucose metabolism and adaptive thermogenesis.

### Human ISG15 deficiency

ISG15 deficiencies in humans are extremely rare and not fatal, while the deficiencies are associated with brain calcification, skin lesions, and mycobacterial hypersensitivity^15,171–173^. ISG15 and USP18 deficiencies have begun to be classified as inherited interferonopathies. Several ISG15-deficient patients suffered from seizures and displayed intracranial calcification, leading to Aicardi-Goutieres-like interferonopathy^15^. USP18 downregulation and a persistent IFN signature have been detected in ISG15-deficient patients^15^. USP18 is degraded via UPS^174^. SKP-Cullin-F-box protein (SCF^SKP2^) accelerates USP18 ubiquitination, thereby leading to its proteasomal degradation. Interestingly, USP18 specifically binds to the second chain of the type I IFN receptor subunit IFN α/β receptor 2 (IFNAR2) and competes with Janus kinase 1 (JAK1) for binding to IFNAR2, which impairs the association of JAK with the IFN receptor and attenuates IFN signaling^82^. Moreover, USP18 is recruited by STAT2 and associates with IFNAR2, thereby displacing JAK1 and suppressing IFN signaling^175^. Notably, the interaction of USP18 with free intracellular ISG15 impairs ubiquitination and proteasomal degradation of USP18, which leads to the prevention of overamplification of IFN signaling and autoinflammation, suggesting that free ISG15-mediated stabilization of USP18 is pivotal for the negative feedback regulation of long-term IFN signaling^15^. However, murine USP18 is not dependent on ISG15 for its stabilization^172^. USP18-deficient patients died shortly after birth owing to massive dysregulation of IFN signaling^176^.

Individuals with inherited ISG15 deficiency show accelerated susceptibility to virulent *Mycobacterium tuberculosis* (*M. tuberculosis*), a condition known as Mendelian susceptibility to mycobacterial disease (MSMD)^89,177^. Although this phenotype was initially ascribed to the extracellular form of ISG15, it was later demonstrated that ISGylation is also involved during *M. tuberculosis* infection in vivo^177^. Patients with six novel mutations in *Isg15* presented skin lesions, and they were managed for dermatologic diseases^171^. In peripheral blood, myeloid cells display the most robust type I IFN signatures. Further, in the affected skin, IFN signatures are detected in the keratinocytes of the epidermis and endothelia, monocytes, and macrophages of the dermis, which collectively defines the specific cells driving dermatologic inflammation and expands the clinical spectrum of ISG15 deficiency to dermatologic presentation. Recently, it has been reported that patients with ISG15 deficiency display an accelerated IFN signature in regulatory T cells (Tregs), suggesting that ISG15 might dictate Treg refractoriness to the effect of IFNs in the course of inflammation^178^.

### ISG15 and its conjugation in cancer

**Fig. 4:**
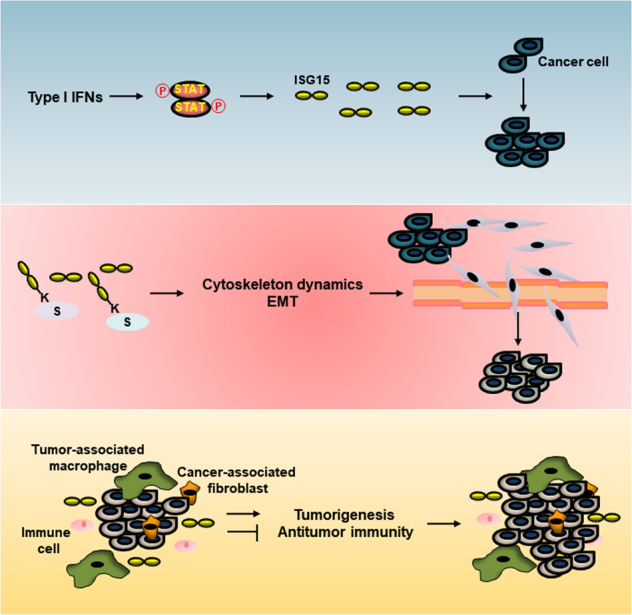
ISG15 and its conjugation in cancer. Upregulation of ISG15 promotes cancer cell proliferation. ISG15 and ISGylation disrupt cytoskeletal architecture and positively regulate EMT, which facilitates the invasion and metastasis of cancer cells. IFNs produced in the tumor microenvironment induce ISG15 expression in tumor-associated macrophages. Secreted ISG15 from TAMs promotes tumor progression and suppresses antitumor immunity.

ISG15 and ISGylation are implicated in cancer (Fig. 4). ISG15 and enzymes involved in ISGylation have been demonstrated to be upregulated in many types of cancer, including melanoma and lung, breast, prostate, and hepatocellular cancers^179,180^. The tumor microenvironment is the environment surrounding tumors, where cells continuously sense danger and damage signals via extracellular and intracellular pattern recognition receptors (PRRs) to coordinate the host immune system^181^. One of the important events is the type I IFN production in response to the activation of specific PRRs, which results in the activation of the JAK-STAT pathway and subsequent induction of ISGs, including ISG15. Furthermore, dysregulation of ISG15 and ISGylation is directly or indirectly linked to the pathogenesis of cancer. Therefore, undoubtedly, many research groups should be encouraged to study why cancer cells upregulate ISG15 and how its upregulation gives an advantage to cancer cell growth. However, ISG15 and ISGylation in tumorigenesis are controversial, likely due to the genetic background of cancers, type of tissues, stage of cancer, and concomitant alterations in particular cancer-related signal transduction pathways. Furthermore, the role of free ISG15 conflicts with the role of its conjugation in terms of cancer pathogenesis, suggesting the necessity of further investigation.

### UBE1L in tumor progression

*Ube1L* expression is downregulated in lung cancer cells, and its upregulation attenuates lung cancer cell growth by inhibiting cyclin D1, which is essential for cell cycle progression^182^. However, it has been demonstrated that UBE1L deficiency neither alters lung cancer progression nor affects the overall survival of *K-ras*^LA2^ lung cancer mice, suggesting that UBE1L is not a tumor suppressor gene in *K-ras*^LA2^ lung cancer mouse model^183^. Additionally, UBE1L does not suppress overall cancer development in a p53 KO cancer model^184^.

UBE1L upregulation in APL upon RA treatment leads to the degradation of oncogenic fusion protein promyelocytic leukemia (PML)-RA receptor α (RARα) (PML-RARα), resulting in the prevention of APL^40^. Therefore, further studies uncovering the molecular mechanisms of UBE1L will provide valuable information about the role of UBE1L in tumor progression.

### Protumoral role of ISG15 and ISGylation

ISG15 upregulation has been demonstrated in several types of cancer, including melanoma and lung, breast, prostate, nasopharyngeal, and oral cancers^13,36,179,182,185–188^. It has been demonstrated that Rab GDP-dissociation inhibitor β (GDI2) ISGylation regulates epidermal growth factor receptor (EGFR) trafficking, which promotes EGFR recycling and sustains AKT signaling^189^. IFNγ-induced ISG15 might affect the response of breast cancer cells to endocrine therapy with fulvestrant or tamoxifen^190,191^. Low levels of p53 and ADP-ribosylation factor (ARF) correlate with high levels of STAT1 and ISG15 in triple-negative breast cancers (TNBCs), which leads to the proliferation and tumorigenicity of TNBCs^187,192^. Tumor samples from TNBC patients with co-inactivated p53 and ARF exhibit upregulation of STAT1 and ISG15, suggesting the oncogenic role of the IFN-STAT1-ISG15 signaling axis. Upregulation of ISG15 and ISGylated proteins in primary tumor cells derived from breast cancer patients has been reported. This finding suggests that ISG15 and ISGylation may be novel breast cancer markers with prognostic significance, which will be valuable for selecting patients and predicting response to the treatment of breast cancer.

ISG15 and ISGylation have been demonstrated to be involved in epithelial–mesenchymal transition (EMT), cell motility, invasion, and metastasis of breast cancer cells. ISG15 impairs cytoskeleton architecture and the formation of focal adhesion in breast cancer cells^179,193,194^. IFNγ upregulates ISG15 and promotes ISGylation in breast cancer cells, which occurs in parallel with changes in the morphology of breast cancer cells^37,195,196^. IFNγ-induced ISGylation of nonmuscle myosin II A (NMIIA) and Ras GTPase-activating-like protein 1 (IQGAP1) is involved in cytoskeletal reorganization^97,194,196^, suggesting the role of ISGylation in the invasion and metastasis of breast cancer cells. ISG15 and ISGylation induced by oncogenic Kirsten-Ras (Ki-Ras) suppress lysosomal degradation of Ki-Ras, which facilitates migration and EMT of breast cancer cells^185^. Endothelial lipase (LIPG) regulates deltex (DTX)-3-like ubiquitin E3 ligase (DTX3L/BBAP)-ISG15 signaling, which modulates protein stability and facilitates the development and metastasis of TNBC, suggesting that the DTX3L/BBAP-ISG15 signaling axis is a potential target for basal-like TNBC therapy^197^.

ISG15 and enzymes involved in ISGylation are upregulated in hepatocellular carcinoma (HCC). ISG15 impairs the interaction of survivin with X-linked inhibitor of apoptosis protein (XIAP) and stabilizes survivin, promoting the proliferation and migration of HCC. ISG15 upregulation in HBV-related HCC tissues has been reported, suggesting ISG15 as a novel prognostic marker for predicting the overall survival of HBV-related HCC patients^198^.

ISG15 and ISGylation have been shown to be upregulated in tumors of two colon cancer patients compared with healthy colon tissues^56^. ISGylation attenuates UPS, which promotes the production of IFN-induced ROS, activates p38 MAP kinase, and upregulates inflammation-related cytokines in macrophages, thereby accelerating intestinal inflammation and colitis-associated colon cancer in mice^199^. ISG15 is upregulated in penta-span transmembrane glycoprotein prominin 1 (PROM1, also known as CD133)-positive colorectal cancer cells compared to PROM1-negative colorectal cancer cells, suggesting the involvement of ISG15 in cancer stemness^200^.

ISG15 upregulation in esophageal squamous cell carcinoma (ESCC) and gastric cancer is associated with clinical outcomes, suggesting that ISG15 could be used as a prognostic marker^201–203^.

Free intracellular ISG15 interacts with Rac1-GDP in membrane protrusions and facilitates Rac1 activity, which induces cell migration and is associated with lymphatic metastasis of oral squamous cell carcinoma (OSCC)^186^. Depletion of autophagy elongation proteins induces ISG15 expression through STAT1 activation, resulting in the acquisition of tumor-associated phenotypes such as cell proliferation, migration, and invasion^204^.

### Antitumoral role of ISG15 and ISGylation

ISG15 in several cancer types of cervix, blood, and ovaries decreases proliferation and increases apoptosis, resulting in tumor suppression^205–207^. ISG15 upregulation in advanced-stage high-grade serous ovarian cancer (HGSOC) leads to an increase in the number of tumor-infiltrating CD8 + lymphocytes, improving median overall survival^207^. ERK ISGylation in HGSOC activates NK cells and CD8 + T lymphocytes, resulting in the inhibition of ovarian cancer progression. 90 K, a tumor-associated glycoprotein, is secreted and associated with CD9/CD82 to induce proteasomal degradation of β-catenin, resulting in the suppression of tumor growth and metastasis. β-catenin degradation is dependent on its ISGylation but not on glycogen synthase 3β (GSK-3β) and Siah/adenomatous polyposis coli (APC)^129^. Epithelial splicing regulatory protein 1 (ESRP1) ISGylation attenuates its degradation, resulting in the suppression of EMT of lung cancer cells^208^. Free ISG15 interacts with HIF1α in a noncovalent manner and prevents the dimerization of HIF1α and downstream signaling^33^.

### ISG15 and its conjugation in cancer immunogenicity

ISG15 secretion from tumor cells, M2 macrophages, or alternatively activated macrophages suppresses adaptive immunity and promotes tumorigenesis, indicating that ISG15 acts as a tumor microenvironment factor in tumor progression and cytotoxic immune suppression. ISG15 secretion from melanoma cells induces E-cadherin expression, which modulates the phenotype of tumor-infiltrating DCs and leads to tumor escape, suggesting the protumoral role of unconjugated ISG15 in cancer immunogenicity^13^. ISG15 can be detected at a high concentration in plasma from patients with ESCC, suggesting the potential of ISG15 as a diagnostic marker of ESCC^209^. In response to type I IFNs produced by PDAC cells, ISG15 is secreted from tumor-associated macrophages (TAMs) and aggravates the tumorigenicity of CSCs by reinforcing CSC self-renewal, invasive capacity, and tumorigenic potential, which suggests key roles of ISG15 in the pathogenesis and progression of CSCs in the PDAC microenvironment^210^. ISG15 is upregulated in the TAMs of primary PDAC tumors resected from patients. TRIM29 depletion facilitates calpain 3-dependent processing of ISG15, which modulates the stability and extracellular release of ISG15 in PDACs and suppresses CSC-like features of PDACs^211^. ISG15 expression is linked to poor prognosis of patients with nasopharyngeal carcinoma (NPC). Furthermore, ISG15 secreted from NPC cells induces macrophages with an M2-like phenotype, which is dependent on the interaction of ISG15 with LFA-1, engagement of SFK signaling, and chemokine (C–C motif) ligand 18 (CCL18) secretion, resulting in tumorigenicity and migration of NPC cells^212^.

UBE1L has been reported to be a tumor suppressor in breast cancer^213^. ISGylation of STAT1 and STAT2 mediate clustering and nuclear relocalization of STAT1 and STAT2 within IFN-induced PML bodies and synergistically promotes the production of chemokine-receptor ligands to attract cytotoxic T cells, thereby suppressing murine breast cancer growth and metastasis.

Vaccination with ISG15 confers regression of HPV-associated tumor burden in mice, providing new insight into the immunomodulatory properties of ISG15 and its potential to serve as an effective immune adjuvant in cancer therapies^214^. The responses induced by vaccination with ISG15 are not dependent on ISGylation. Moreover, ISG15 produced at the vaccination site facilitates the vaccine-specific cytotoxic T lymphocyte response, suggesting that ISG15, as an alarmin, induces tissue alert via extracellular matrix remodeling, myeloid cell infiltration, and inflammation^215^.

### ISG15 and its conjugation in therapies for cancer

ISG15 and ISGylation are implicated in a variety of therapies for cancer^40,110^. In concordance with their roles in the DNA damage response, ISG15 and ISGylation might be key determinants of therapeutic efficacy. Further, the identification of IRDS genes pinpoints ISG15 as an essential sign of resistance to DNA-damaging therapies, indicating an interplay between the innate immune system and therapeutic response. Therefore, not only elucidating the mechanisms by which ISG15 and ISGylation modulate sensitivity or resistance to therapies but also investigating the possibility that ISG15 and ISGylation can serve as indicators for the selection of therapy could be promising for improving cancer patient survival.

### Sensitivity to therapies for cancer

ISG15 downregulation decreases the sensitivity of breast cancer cells to camptothecin. ISG15 is upregulated in irinotecan-sensitive tumors from gastric cancer patients compared with irinotecan-resistant tumors^203,216^. Clioquinol and mefloquine used to treat leukemias and myelomas upregulate ISG15 and promote apoptosis through the modulation of nuclear factor kappa-light-chain-enhancer of activated B cells (NF-κB) signaling^206^. ISG15 overexpression downregulates ATP binding cassette subfamily C member 2 (ABCC2) and facilitates sensitivity to cisplatin in cisplatin-resistant ovarian cancer cells^116^. Mechanistically, hnRNPA2B1 ISGylation inhibits its recruitment to ABCC2 mRNA, suppressing ABCC2 translation.

Doxorubicin-induced ΔNp63α ISGylation not only facilitates the transactivity of proapoptotic p53 family members but also suppresses the oncogenic ability of ΔNp63α, suggesting the contribution of ISGylation to therapeutic efficacy^4^. Moreover, DNA-damaging therapies induce p53 ISGylation, which promotes the expression of p53 target genes as well as its own gene and suppresses cell growth and tumorigenesis^217^. In colorectal cancer cells, ISG15 upregulation by the DNA-demethylating agent 5-aza-2-deoxycytidine (5-AZA-CdR) that induces viral mimicry and targets colorectal cancer-initiating cells (CICs) indicates the role of ISG15 in the modulation of therapeutic efficacy^218^.

### Resistance to therapies for cancer

ISG15 is associated with therapeutic resistance, although the association remains elusive. ISG15 upregulation is associated with gemcitabine resistance in pancreatic cancer cells^219^. Treatment with ISG15 peptides suppresses primary and metastatic mammary tumor burden in mice^220^. ISG15 is upregulated following trametinib treatment in colon cancer cells and suppresses the anticancer effect of trametinib, suggesting combined targeting of ISG15 and mitogen-activated protein kinase kinase (MEK) as a promising therapeutic strategy for colon cancer treatment^221^.

ISG15 is one of the IRDS gene products^110^. IRDS has been characterized as a gene signature for resistance to DNA-damaging therapies, suggesting that data regarding IRDS significantly improves outcome prediction when combined with standard markers, risk groups, or other genomic classifiers^109,110^. ISG15 drives chemotherapy and radiation resistance of breast cancer cells in a process involving paracrine and juxtacrine communications between stroma and breast cancer cells^113^. ISG15 upregulation in breast cancer correlates with not only poor response to chemotherapy and radiotherapy but also subsequent unfavorable prognosis^220^. ISG15 upregulation in NPC is linked to pluripotency-associated gene expression and resistance to DNA-damaging therapies.

## Conclusions

In contrast to the constitutive expression of some UBLs, ISG15, and enzymes that catalyze ISGylation are induced by a large variety of cues, indicating that ISG15 and ISGylation are tightly fine-tuned. Despite the early classification of ISG15 as a UBL, it was not until 2002 when the first targets for ISGylation were identified. Since then, proteomic studies have identified a large variety of target proteins for covalent or noncovalent associations with ISG15 not only in normal contexts but also in disease settings. Given that one or multiple positions for ISGylation on target proteins could represent cues for signal recognition, integration, and transduction in collaboration with ISG15 interactors in a topology-dependent manner, further investigation of the topologies of ISG15 and topology-specific downstream ISG15 receptors to decode and translate ISG15 and ISGylation into biological functions will provide mechanistic insights into the multifaceted roles of ISG15 and ISGylation.

Given that ISG15 is significantly induced by IFNs and that the tumor stroma is infiltrated by immune cells, it is conceivable that immune cells provide the source of IFNs, triggering the signal for robust induction of ISG15 in cancer cells. Therefore, it is essential to elucidate not only the multitude of cellular processes in cancer immunogenicity in which ISG15 and ISGylation are implicated but also the communication between tumor cells and their microenvironment for the improvement of the efficacy of cancer therapies.
